# Society of Physicians and Surgeons of Southwestern Iowa

**Published:** 1877-07

**Authors:** 


### SOCIETY OF PHYSICIANS AND SURGEONS OF SOUTHWESTERN IOWA.

In pursuance of a call signed by the regular physicians in Adams, Adair, Clarke, Ringgold, Taylor and Union counties, Drs. Howe, Griggs, Scroggs, Beebe, Reynolds, Ran, T. Johnston; Ryno, Wilson, Given, Torrey, Vance and Christie met in the office of Drs. Christie and Torrey at Creston, Iowa, on the 1st day of June, 1876, for the purpose of organizing a medical society.

The constitution adopted named the society, The Society of Physicians and Surgeons of Southwestern Iowa.

Art. 3d provides that the members of this society shall consist of physicians and surgeons residing in the counties mentioned, who are authorized to practice medicine and surgery by any medical college accredited by the American Medical Association; and of such persons as are engaged in the regular practice of medicine, and are in good repute.

Art. 10th says the meeting of this society shall be quarterly and to be held on the first Tuesday of June, September, December and March of each year.

The following officers were elected for the ensuing year: J. B. Wilson, President; J. D. Reynolds, Vice President; W. H. Christie, Secretary; J. E. Howe, Treasurer.

Drs. J. P. Scroggs, R. C. Gregg, J. E. Howe, H. A. Given and L. S. Groves, Censors.

The President appointed committees to report at next meeting, which was to be held the first Tuesday in September.

Owing to an inclement state of the weather on the regular day of meeting, the society did not meet until Sept. 12.

The following papers were read:

Malarial Bronchitis, by Dr. Gregg, chairman of the committee on practice.

Drs. Christie, Reynolds, Howe and Wilson made remarks upon a continued type of malarial fevers, presenting in the latter stage meningeal complications.

Drs. Wilson and Christie had not seen this fatal complication in any cases where quinia had been properly administered in the onset of the fever.

Dr. Christie gave quinia largely early in the treatment, and brom. potass. with spts. nit. aether; later, dropped the quinia and continued potassic bromide combined with ergot and cannabis Ind., with but little advantage. He used also diluted hydrochloric acid and chlorate of potash.

Dr. Wilson used large doses of quinia early, and where there was a tendency to brain trouble, bromide of potash in large quantity.

Dr. Christie reported a paper upon corporeal endometretis.

Dr. Torrey, one upon cholera infantum.

Dr. Reynolds, as one of a committee on materia medica, reported his manner of using verat. viride, aconite and gelseminum.

Dr. Nance, as the other member of the committee, read a paper upon rational therapeutics.

Dr. Given, as committee upon obstetrics, read a paper upon labor, and reported a case where decapitation was necessary to delivery.

It was an arm presentation, and a midwife had seized the arm and pulled the child down.

Dr. Wilson having been appointed essaysist at the other meeting, read a paper upon "rational and progressive medicine."

The December meeting was held at Creston.

The following papers were read:

Drs. Beebe and Christie, as committee, reported upon Surgery. Dr. B. reported a case of strangulated hernia which had been mistaken for an abscess, and poulticed with all sorts of fomentations. He was finally called; operated, patient died. Dr. Christie reported on fractures and hydrocele, and the methods he adopted in their treatment.

Dr. Horne, as committee upon practice, reported upon autumnal fevers.

Dr. Nance read a paper as essayist upon originality in medicine.

Dr. J. B. Wilson read a paper upon syphilis. The report did not endorse the dualistic theory, and the treatment was anti-mercurial. Iod. of potassium was relied upon in large doses.

Dr. Nance offered the following resolution:

*Resolved*, That we as a society recognize the CHICAGO MEDICAL JOURNAL AND EXAMINER as the medical exponent of the society, and that the President of the society appoint a committee, of whom the Secretary shall be one, which shall refer any meritorious reports, essays or papers to the above journal for publication.

The next meeting of the society was appointed at Creston.

Dr. Spooner reported a paper upon obstetrics.

Dr. Given reported a paper upon nervous diseases.

Drs. Torrey and Gregg reported upon materia medica.

Dr. Nance reported on diphtheria.

Although there were not many papers read, yet the character of the papers and reports were unusually interesting, and brought out reports of cases under the different heads.

The next meeting is to be held in Mt. Ayr, on the first Tuesday in June, 1877.

